# Seagrass can mitigate negative ocean acidification effects on calcifying algae

**DOI:** 10.1038/s41598-018-35670-3

**Published:** 2019-02-13

**Authors:** Ellie Bergstrom, João Silva, Cíntia Martins, Paulo Horta

**Affiliations:** 1 School of Environment & Science and Australian Rivers Institute – Nathan Campus, Griffith University, 170 Kessels Road, Brisbane, Nathan, Queensland 4111 Australia; 2 CCMar - Centre of Marine Sciences, University of Algarve, Campus of Gambelas, 8005-139 Faro, Portugal; 3 Department of Ecology and Zoology, Center for Biological Sciences, Federal University of Santa Catarina, 88010-970 Florianópolis, SC Brazil

## Abstract

The ultimate effect that ocean acidification (OA) and warming will have on the physiology of calcifying algae is still largely uncertain. Responses depend on the complex interactions between seawater chemistry, global/local stressors and species-specific physiologies. There is a significant gap regarding the effect that metabolic interactions between coexisting species may have on local seawater chemistry and the concurrent effect of OA. Here, we manipulated CO_2_ and temperature to evaluate the physiological responses of two common photoautotrophs from shallow tropical marine coastal ecosystems in Brazil: the calcifying alga *Halimeda cuneata*, and the seagrass *Halodule wrightii*. We tested whether or not seagrass presence can influence the calcification rate of a widespread and abundant species of *Halimeda* under OA and warming. Our results demonstrate that under elevated CO_2_, the high photosynthetic rates of *H. wrightii* contribute to raise *H. cuneata* calcification more than two-fold and thus we suggest that *H. cuneata* populations coexisting with *H. wrightii* may have a higher resilience to OA conditions. This conclusion supports the more general hypothesis that, in coastal and shallow reef environments, the metabolic interactions between calcifying and non-calcifying organisms are instrumental in providing refuge against OA effects and increasing the resilience of the more OA-susceptible species.

## Introduction

Seagrass meadows and calcifying algae beds are benthic communities that play unique roles in the removal, storage and release of carbon from seawater, via photosynthesis and/or calcification^1^. Coastal communities are metabolically responsible for 85% of the organic carbon and 45% of the inorganic carbon (C_i_) buried in coastal sediments^2–4^. CO_2_ is essential to photosynthesis, yet its increase in seawater reduces pH and carbonate ions, threatening the calcification process^5^. However, these ecosystems naturally experience large vertical and horizontal variations in abiotic parameters, namely pCO_2_ and temperature^6^, that can vary from 400 to 10,000 µatm^2^ and 15 to 30 °C^7^, respectively. Research has suggested that exposure to natural fluctuations alongside possession of phenotypic plasticity may help organisms and populations to resist or acclimate to novel anthropogenic conditions^6,8^.

Little is known about the existing interactions between calcifying and non-calcifying primary producers under OA and temperature rise. Whether it is via alteration of seawater chemistry, allelopathy or other molecular signaling, neighboring marine plants interact by influencing each other’s metabolisms^9^. Changes in benthic macrophyte communities are projected for the future^10^ where altered competition dynamics between fleshy and calcifying algae already have been shown to drive ecosystem shifts under elevated CO_2_ conditions^11^. The current incomplete understanding of these interactions and the consequent mechanisms that drive ecosystem changes limit our ability to make realistic predictions for the effects of OA and warming on future community structure.

Seagrasses can act as buffers to OA by absorbing large quantities of CO_2_ and increasing the pH of seawater^12–14^. Diel pH fluctuations of 0.7–1 pH due to the photosynthesis and respiration of seagrass beds, have been reported in different locations^13,15^. Increased ambient pH levels during the day can become locally significant to the point where they have a positive effect on the calcification of co-occurring calcifying algae^12,13^. However, since oceanic conditions are rapidly changing, information is needed about how the presence of seagrasses will affect calcifying algae responses under OA and temperature rise.

Most of the studies regarding the impact of global stressors evaluate the isolated responses of primary producers, using unifactorial models or eventually considering the combined role of OA and temperature rise in the fitness of a specific and isolated biological indicator^16^. Thus far, the expected trend for seagrasses is neutral to positive physiological responses to OA^1^, yet the magnitude of change and affinity for DIC species varies^17,18^. The isolated effects of temperature and CO_2_ on the seagrass genus *Halodule* Endlicher^17,19,20^ and their isolated and combined effects on the calcifying green algae genus *Halimeda* J.V. Lamouroux have been widely addressed^21–28^. The general consensus of OA studies on *Halimeda* indicates negative to neutral calcification responses and neutral to positive photosynthetic responses to CO_2_-enriched seawater, due to species specificity^22,24,26–33^.

To date, two studies have considered the effects of seagrass-calcifying algae interactions under ambient conditions^12,13^, but none have addressed how OA and temperature rise influence these ecophysiological interactions. The species-specific nature of the isolated responses emphasizes the necessity to conduct studies that address OA and temperature rise together in order to better understand the mechanisms behind the presence/absence of interactions between these drivers. Short-term mesocosm experiments that simulate rapid heat waves and acidification, as observed in different regions, are fundamental tools to predict complex ecosystem interactions^34,35^. It is also necessary to introduce realism in these simulations by representing the high-frequency semidiurnal or diurnal variability that dominates coastal or shallow environments^36^. Recent studies reveal that under OA, net photosynthesis of the kelp *Ecklonia radiata* was almost 50% lower when pH fluctuated than when it was static^37^. This natural variability imposes particularities that can limit or stimulate primary production and must be reproduced in order to properly simulate the predictable future scenarios.

Here we investigate the effects of OA on the photosynthesis and calcification of the seagrass *Halodule wrightii* and the green alga *Halimeda cuneata* via a full factorial mesocosm design. We simulate OA and warming by exposing the calcifying alga and the seagrass to the following four combinations of ambient and elevated pCO_2_ and temperature: 28 °C & 320 µatm, 28 °C & 822 µatm, 30 °C & 320 µatm and 30 °C & 822 µatm. Most importantly, we examine the degree to which the photosynthetic carbon uptake of *H. wrightii* influences seawater chemistry under OA through short-term incubations. We aim to determine whether this can act as a metabolic feedback on the photosynthesis and calcification of *H. cuneata*, considering that these two species of macrophytes coexist in the shallow tropical waters off the Brazilian coast^38^. We hypothesize that *H. wrightii* is capable of using the excess DIC resulting from OA to increase its photosynthetic activity. In mitigating the effects of OA on seawater chemistry, we hypothesize that the negative effects of OA on the calcification rate of *H. cuneata* may consequently be ameliorated.

**Table 1:** ANOVA results showing (1) the effect of elevated CO_2_ and seagrass presence on the calcification and gross primary production (GPP) for *H. cuneata*, (2) the effect of elevated CO_2_ on GPP for *H. wrightii* and (3) the effect of temperature on the calcification and GPP of both primary producers (highlighted in gray).

Source of variation		Calcification			GPP		
*H. cuneata*	df	MS	*F*	*p*	MS	*F*	*p*
CO_2_ treatment (CO_2_)	1	0.61	7.06	**0.017**	0.99	0.85	0.370
Seagrass presence (S.P.)	1	0.44	5.10	**0.038**	31.24	26.69	**0.000**
S.P.*CO_2_	1	0.11	1.25	0.281	1.83	1.57	0.229
Temperature	1	0.03	0.15	0.706	0.03	0.01	0.918
*H. wrightii*							
CO_2_	1	—	—	—	40.64	1.09	0.321
Temperature	1	—	—	—	40.17	0.98	0.351

Significance was considered when *p* < 0.05.

**Figure 1:**
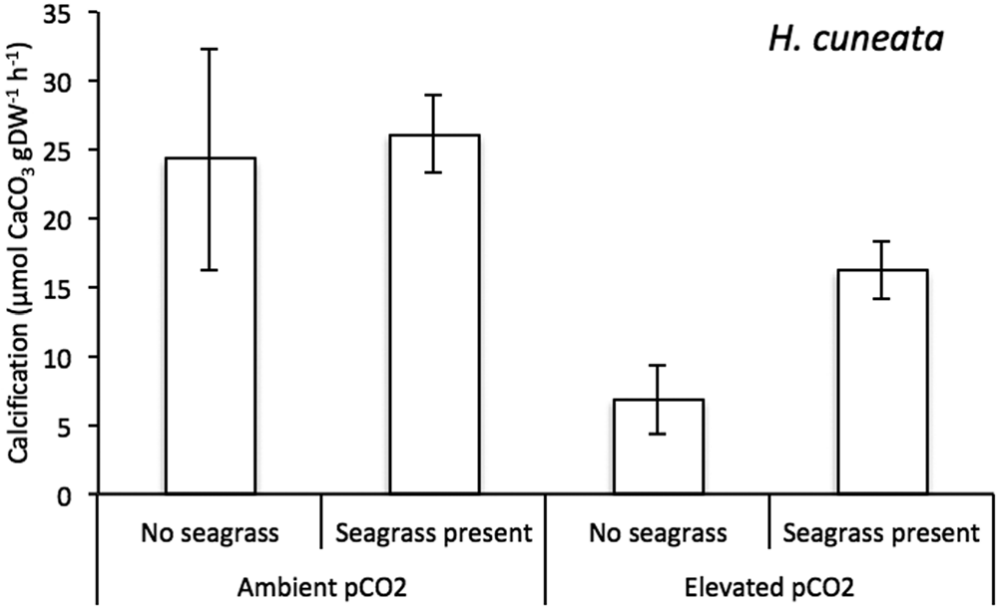
Mean calcification responses of *H. cuneata* (n = 5) ± SEM in the presence & absence of seagrass, under ambient (380 µatm) and elevated (822 µatm) pCO_2_ levels. Values were normalized to the dry, decalcified weight (DW) of only *H. cuneata*.

## Results

After exposure to treatments for 10 d, short-term incubations (illustrated in Supplementary Fig. S1) of *H. cuneata* and *H. wrightii* separately, as well as together, were conducted in order to understand their physiological responses to OA. We also sought to obtain the magnitude of the effect that the primary production of these macrophytes, particularly the seagrass, has on surrounding seawater chemistry under the stress of OA. The ultimate objective was to determine whether biologically altered seawater might be sufficient enough to mitigate the effects of OA on *H. cuneata* calcification.

**Figure 2:**
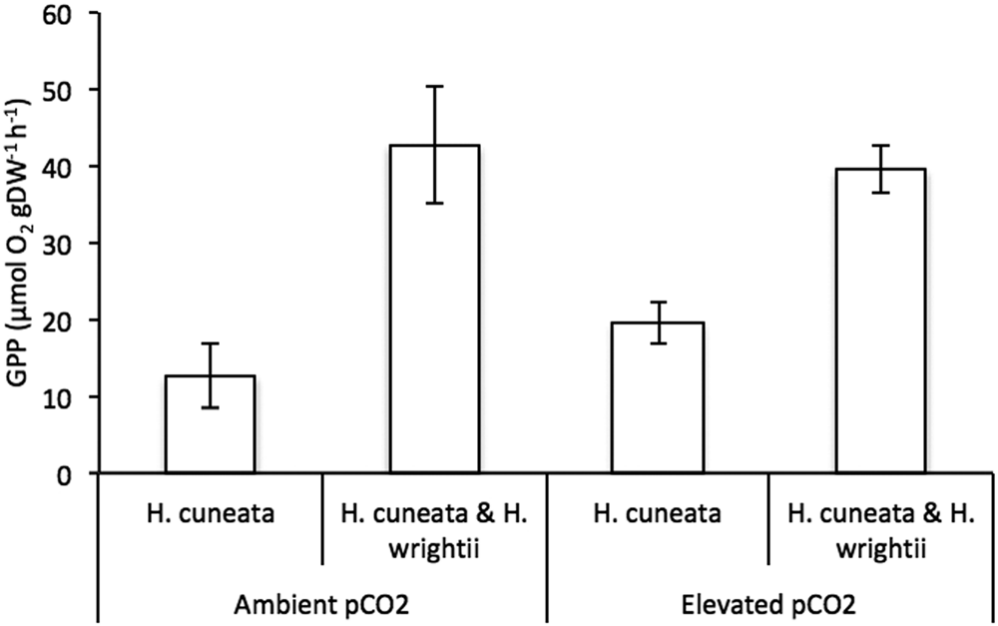
Mean gross primary production (GPP) responses of *H. cuneata* (n = 5) ± SEM and *H. cuneata* & *H. wrightii* together (n = 5) ± SEM, under ambient (380 µatm) and elevated (822 µatm) pCO_2_ levels. Values were normalized to the dry, decalcified weight (DW) of the species present.

**Figure 3:**
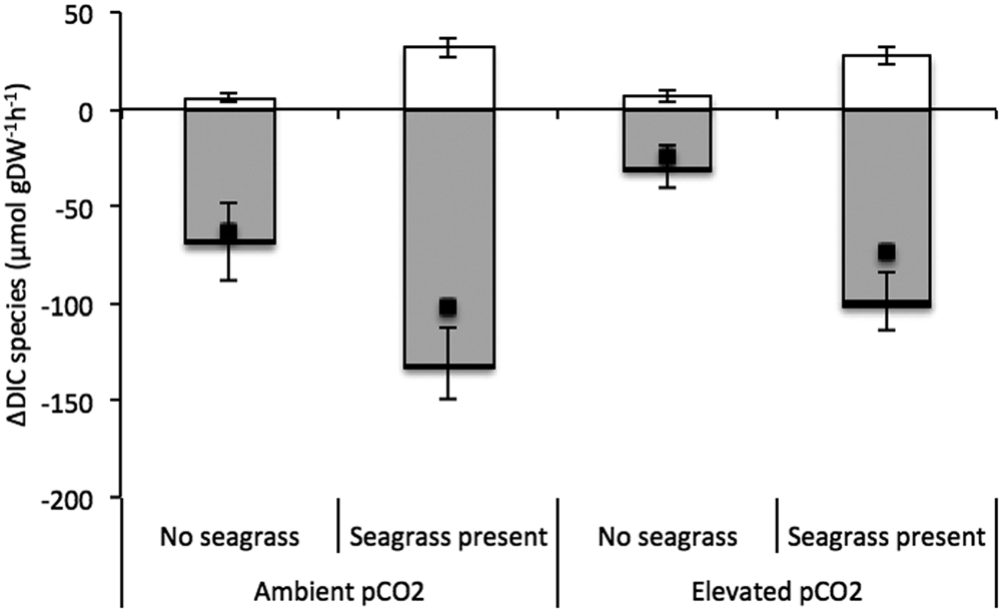
Changes in DIC species (HCO_3_^−^, CO_2_ & CO_3_^2−^) ± SEM during the incubation of *H. cuneata* with and without seagrass present, at ambient (380 µatm) and elevated (822 µatm) pCO_2_ levels. Negative changes indicate consumption of the DIC species. White bars refer to ΔCO_3_^2−^, grey bars refer to ΔHCO_3_^−^ and black bars refer to ΔCO_2_. Total DIC (ΔDIC) is the sum of the change in all DIC species and is represented by the black scattered points. When no seagrass was present, values were normalized to the dry, decalcified weight (DW) of *H. cuneata* (n = 5). When seagrass was present, values were normalized to the sum of the dry, decalcified weight of *H. cuneata* + *H. wrightii*.

*H. cuneata* and *H. wrightii* fared differently under OA conditions. While the calcifying alga experienced negative physiological consequences (Fig. 1), the seagrass showed a neutral response (Fig. 2, Table 1). We report a significant effect of CO_2_ enrichment on *H. cuneata* calcification (*p* = 0.017, Table 1), causing it to suffer a 72% decrease under elevated pCO_2_. Simultaneously, we observed a shift in carbonate chemistry when *H. cuneata* was incubated in the elevated pCO_2_ treatment, where HCO_3_^−^ decreased by 56% and total DIC, by 60%, but carbonate and CO_2_ remained the same (Fig. 3, Table 2).

**Table 2:** Mean values of calcification, gross primary production (GPP) and the changes in bicarbonate (ΔHCO_3_^−^), carbon dioxide (ΔCO_2_), carbonate (ΔCO_3_^2−^), total DIC (ΔDIC), aragonite saturation state (ΔΩ_Ar_) and pH (ΔpH) ± SEM for *H*.

Species	CO_2_	Seagrass presence	Calcification (µmol CaCO_3_ g^−1^h^−1^)	GPP (µmol O_2_ g^−1^h^−1^)	ΔHCO_3_^−^ (µmol g^−1^h^−1^)	ΔCO_2_ (µmol g^−1^h^−1^)	ΔCO_3_^2−^ (µmol g^−1^h^−1^)	ΔDIC (µmol g^−1^h^−1^)	ΔΩA_r_ (g^−1^h^−1^)	ΔpH
*H. cuneata*	Ambient	—	24.29 ± 8.07	12.61 ± 4.10	−68.28 ± 20.08	−0.74 ± 0.16	5.43 ± 2.11	−63.58 ± 19.44	0.08 ± 0.03	0.08 ± 0.04
	Elevated	—	6.85 ± 2.49	19.45 ± 2.65	−29.87 ± 10.65	−1.46 ± 0.71	6.12 ± 2.98	−25.20 ± 8.76	0.09 ± 0.05	0.07 ± 0.02
	Ambient	+	26.09 ± 2.84	42.72 ± 7.58	−131.25 ± 18.09	−1.63 ± 0.48	31.47 ± 4.66	−101.42 ± 13.98	0.49 ± 0.07	0.21 ± 0.05
	Elevated	+	16.15 ± 2.09	39.46 ± 3.13	−99.13 ± 14.62	−2.53 ± 0.64	27.23 ± 4.73	−74.43 ± 10.13	0.42 ± 0.07	0.25 ± 0.06
*H. wrightii*	Ambient	n/a	—	677.48 ± 169.87	−1097.94 ± 237.31	−20.06 ± 7.7	313.53 ± 39.11	−804.47 ± 206.7	4.85 ± 0.6	0.16 ± 0.03
	Elevated	n/a	—	464.11 ± 60.23	−1484.45 ± 163.84	−33.0 ± 9.21	490.06 ± 71.52	−1027.38 ± 117.81	7.57 ± 1.11	0.27 ± 0.05

*cuneata* and *H. wrightii*. These result from ambient and elevated pCO_2_ levels of 380 and 822 µatm, respectively and the absence/presence of seagrass (for *H. cuneata* only).

We did not detect a significant effect of CO_2_ on the gross primary production (GPP) of either *H. cuneata* (*p* = 0.370) or *H. wrightii* (*p* = 0.321) when incubated separately (Table 1), yet for *H. cuneata* there was an increasing trend (Fig. 2, Table 2). When *H. cuneata* and *H. wrightii* were incubated together, the resulting GPP also did not differ from ambient to elevated pCO_2_ (Table 2), but the overall values were clearly driven by *H. wrightii* production. The GPP of *H. wrightii* was about 53 times higher than that of *H. cuneata* at ambient conditions and 23 times higher under elevated CO_2_ (Table 2).

**Figure 4:**
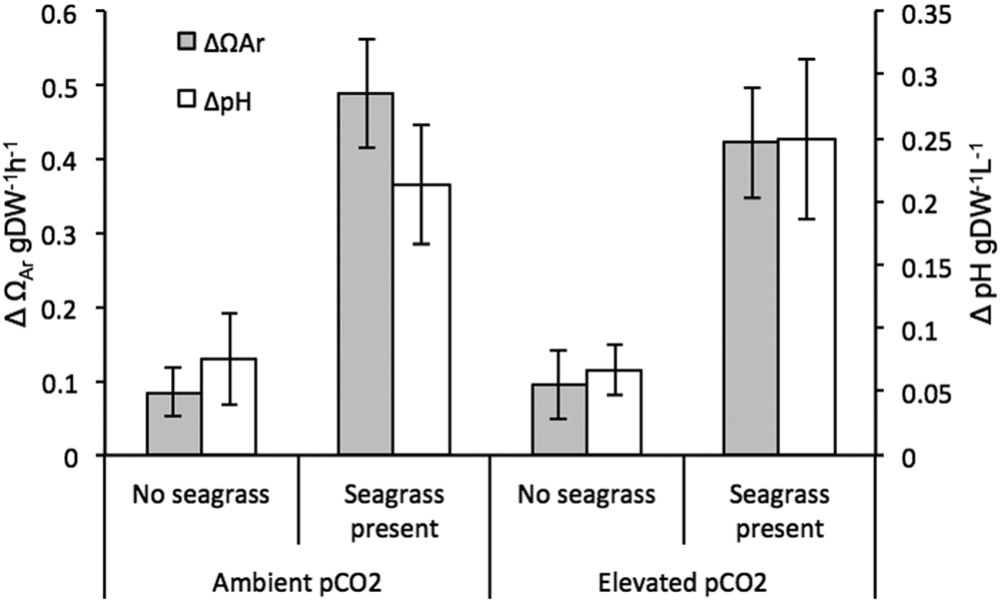
Changes in aragonite saturation state (ΔΩAr; grey bars) and pH (ΔpH; white bars) of seawater ± SEM resulting from the incubation of *H. cuneata* in two CO_2_ treatments (380 µatm & 822 µatm) and in the absence/presence of seagrass. When no seagrass was present, values were normalized to the dry, decalcified weight (DW) of *H. cuneata* (n = 5). When seagrass was present, values were normalized to the sum of the dry, decalcified weight of *H. cuneata* + *H. wrightii*.

We observed significant changes in seawater chemistry (Table 3) resulting from the incubation of *H. cuneata* and *H. wrightii* separately and together. It’s worthy to note, however, that observed shifts that we attribute to an organism’s metabolism also reflect the natural chemical equilibrium change that occurs following biological inorganic carbon uptake. The challenge of teasing apart the biological effect and the equilibrium change warrants relative interpretation of DIC uptake. Surprisingly, *H. wrightii* did not increase DIC uptake under OA, showing no significant differences in total DIC consumption between ambient and elevated CO_2_ (*p* = 0.356, Table 3). However, due to its comparably higher GPP, *H. wrightii* still took up 40 times more total DIC than *H. cuneata* under elevated CO_2_ (Table 2). Consequently, when incubated alone under elevated CO_2_, *H. wrightii* was able to metabolically increase seawater pH by 0.27 ± 0.05 units and aragonite saturation state (Ω_Ar_) by 7.57 ± 1.11 units (Table 2). In contrast, under the same conditions, *H. cuneata* only increased seawater pH by 0.08 ± 0.04 and Ω_Ar_ by 0.08 ± 0.03 (Fig. 4, Table 2). When incubated with *H. cuneata*, seagrass presence was a significant factor in determining the evolution of HCO_3_^−^, CO_3_^2−^ and total DIC as well as ΔΩ_Ar_ and ΔpH in seawater (Table 3). We were not able to quantitatively separate the photosynthetic rates of the alga and the seagrass when they were incubated together. So in order to estimate the effect of the seagrass, we relied on the magnitude of metabolic change for which the seagrass was solely responsible when incubated alone, as mentioned previously.

**Table 3:** Results from the ANOVA used to test the effect of elevated CO_2_ and seagrass presence on DIC species evolution for *H. cuneata* incubations and the ANOVA used to test the effect of elevated CO_2_ on DIC species evolution for *H. wrightii* incubations.

Source of variation		ΔHCO_3_^−^			ΔCO_2_			ΔCO_3_^2−^			ΔDIC			ΔΩA_r_			ΔpH		
*H. cuneata*	df	MS	*F*	*p*	MS	*F*	*p*	MS	*F*	*p*	MS	*F*	*p*	MS	*F*	*p*	MS	*F*	*p*
CO_2_ treatment (CO_2_)	1	9140	7.28	**0.015**	4.59	2.83	0.111	82	1.37	0.258	7126	7.65	**0.013**	0.02	1.38	0.256	0	0.25	0.621
Seagrass presence (S.P.)	1	18490	14.72	**0.001**	6.43	3.97	0.063	2386	40.0	**0.000**	8040	8.63	**0.009**	0.57	39.0	**0.000**	0.09	13.46	**0.002**
S.P.*CO_2_	1	62	0.05	0.827	0.24	0.15	0.704	113	1.89	0.187	11	0	0.917	0.03	1.86	0.191	0	0.06	0.805
*H.wrightii*																			
CO_2_	1	331964	1.92	0.209	372.19	1.08	0.333	69252	4.02	0.085	110422	1.0	0.356	16.44	3.95	0.087	0	2.81	0.138

Significance was considered when *p* < 0.05.

The seagrass was able to mitigate OA and significantly impact the alga’s physiology. We report significant effects of seagrass presence on *H. cuneata* calcification (*p* = 0.038), but no interactive effect was found between CO_2_ treatment and the latter (*p* = 0.281, Table 1). When *H. wrightii* and *H. cuneata* were incubated together at ambient pCO_2_, there was interestingly no observed change in calcification. However, at elevated pCO_2_, the metabolic interaction between the two mitigated the negative impact of OA and the calcification rate of *H. cuneata* was reduced by only 34% (as opposed to 72% when alone; Fig. 1, Table 2).

During the experimental period, the ambient CO_2_ treatment had a mean pCO_2_ of 380 µatm ± 7 SEM and a mean pH of 8.197 ± 0.006 SEM and for the elevated CO_2_ treatment, a mean pCO_2_ of 822 µatm ± 16 SEM and a mean pH of 7.923 ± 0.007 SEM (Table 4). pH fluctuated throughout the experiment due to natural variation from the adjacent reef, as shown in Supplementary Fig. S2. Mean A_T_ was 2278 ± 2 (ambient) and 2280 ± 7 (elevated; Table 4). There was no effect of CO_2_ treatments on mean seawater TA (*p* = 0.723). The ranges of these parameters as well as the remaining physicochemical characterisation of seawater are found in Table 4. Temperature showed no significant effects or interactions on all descriptors (Table 1). Supplementary Fig. S3 shows the average PAR values observed throughout the day during the experimental period.

**Table 4:** Seawater characterisation of the two CO_2_ treatments.

	Ambient CO_2_ treatment	Elevated CO_2_ treatment RCP 6.0
S	37.7 ± 0.3	37.6 ± 0.4
T (°C)	28.99 ± 0.09	29.04 ± 0.09
pH (NBS)	8.197 ± 0.006 (7.990–8.395)	7.924 ± 0.007 (7.685–8.145)
TA (µmol kgSW^−1^)	2278 ± 2	2280 ± 7
pCO_2_ (µatm)	380 ± 7 (203–666)	822 ± 16 (431–1486)
Phosphate (µM)	0.17 ± 0.06	
Nitrate & Nitrite (µM)	1.22 ± 0.07	
Ammonium (µM)	1.64 ± 0.14	

Shown are the means ± SEM (and ranges for pH and pCO_2_) for each parameter.

## Discussion

The presence of the seagrass *H. wrightii* mitigated the negative effect of OA on the calcification of the alga *H. cuneata*. This is the first study to confirm that under elevated CO_2_ concentrations, seagrass is still capable of maintaining comparatively high photosynthetic rates, and in turn, creating seawater conditions that are conducive to the calcification of sympatric, and otherwise ill-fated, calcifying algae. On their own, *H. cuneata* and *H. wrightii* responded differently to OA. The alga suffered decreased calcification and both the alga and the seagrass showed no significant photosynthetic response. It has widely been shown that calcifying organisms respond negatively to OA, whereas fleshy plants respond neutrally or positively^1,39^. The difference in the magnitude of the metabolic effect that each organism had on the surrounding seawater was substantial, where the dominant effect of *H. wrightii* played to the calcifying alga’s advantage under OA. It is worthy to note that the range of temperature initially tested (28–30 °C) to simulate ocean warming need not be considered a frontrunner threat to *H. cuneata* and *H. wrightii* photosynthesis and calcification due to the absence of an observable effect of temperature on these processes.

One of the more interesting findings of our study is the apparent asymmetry between photosynthetic and calcification responses to OA in *H. cuneata*. The calcified macroalgae showed signs of physiological stress, seeing as we observed a substantial decrease (72%) in its calcification. On the other hand, the photosynthetic response was not significant due to variation within treatment, however the fact that there was a 54% increase is worthy of consideration. These two processes occur side by side at the cellular level. Calcification occurs in intercellular spaces (inter-utricle spaces, or IUSs), which are separated from bulk seawater by a layer of utricles where photosynthesis is concentrated^40^. DIC uptake during photosynthesis is found to increase the pH of IUSs and calcium carbonate precipitation is favoured. Conversely, calcification produces H^+^ and CO_2_, which balance the change in pH and CO_2_ concentration produced by photosynthesis^40^. Due to the close proximity of these processes, under ambient conditions, calcification is reported to be closely coupled to photosynthesis in the genus *Halimeda*^41,42^. Thus, when photosynthesis increases, calcification is expected to increase, and vice versa. However, when we elevated CO_2_ in this study, calcification was compromised despite an increasing trend in GPP (Table 2), which suggests that there may be a certain degree of independence between these processes.

The understanding of this uncoupling lies in the carbonate chemistry dynamics of the location where photosynthesis and calcification intersect, the IUSs. Ultrastructure data suggests that the structure and size of utricles and IUSs in *Halimeda* may help to explain the carbonate chemistry of the IUSs and thus, OA responses^24,41^. Peach *et al*. 2017 found an inverse relationship between diffusive pathway type and mineral content, where species with longer utricles and thinner pathways contained more aragonite than those with shorter utricles and wider pathways. Morphological parameters were not one of our response variables, but based on our results and previously established calcifying mechanisms for *Halimeda*^40,43^ and aquatic plants^44^, we suggest that the diffusive pathway of *H. cuneata* permits corrosive bulk seawater to replenish the IUSs at a much faster rate than it can be biologically-regulated, thus partially inhibiting calcification^45^. We also suggest that dissolution may be contributing to the low pH and DIC-rich environments in the IUSs. The degree to which the alga may be experiencing dissolution is also not evident, due to the difficulty of disentangling the effects of dissolution and decreased calcification in OA studies^42,46^. The alga may be using DIC directly from dissolution as substrate for photosynthesis, thus explaining why DIC from bulk seawater was in lesser demand, shown by the 60% decrease in DIC consumption. However, the GPP of the alga was not capable of ameliorating the imbalance in carbonate chemistry of IUSs enough to stimulate calcification, thus our data supports the hypothesis that photosynthesis and calcification become uncoupled under OA^1^. Nonetheless, we cannot be certain of the source of DIC for this increasing trend in photosynthesis, nor the reason that the alga was unable to further increase GPP. Further research on this species using microsensors would be essential to ascertain these unknown thresholds that explain the apparent disparity between photosynthetic and calcification responses to OA^46^.

We initially expected an increase in the GPP of *H. wrightii*, since additional CO_2_ substrate is expected to stimulate primary production in fleshy marine plants. However, our results show that there was no significant change in photosynthesis at elevated CO_2_. Recent meta-analyses report neutral to positive photosynthetic responses of seagrasses to elevated CO_2_^1,47^, which maintains our results within the range of expected responses. In addition, a study that exposed tropical *H. wrightii* to reduced pH observed an increase of only 20% in its photosynthetic rate, followed by a prominent plateau that was attributed to a preference for HCO_3_^−^ use^17^. In the same study, the absence of a change in photosynthesis in *H. wrightii* after the exposure to acetazolamide (AZ), an inhibitor of carbonate anhydrase (CA), a common enzyme that aids in conversion of HCO_3_^−^ to CO_2_, indicated that this species has an alternate and more efficient mechanism for HCO_3_^−^ use when compared to other seagrasses^17^. Our observations likely indicate that *H. wrightii*’s neutral response to OA is due to the efficiency of its mechanism of HCO_3_^−^ use. Due to its much higher photosynthetic rate (464 ± 60 (SEM) µmol O_2_ gDW^−1^ h^−1^), when compared to *H. cuneata* (19.5 ± 2.7 (SEM) µmol O_2_ gDW^−1^ h^−1^) at elevated pCO_2_, *H. wrightii* removes 40 times more DIC from seawater, thus increasing the pH^48^, CO_3_^2−^ availability and aragonite/calcite saturation states^49^. Although the seagrass did not increase its GPP, its capacity to biologically alter its surrounding seawater chemistry was enough to influence the metabolism of coexisting *H. cuneata*. We did not quantify the density of the studied *Halodule* bed, which is a factor that is shown to affect the magnitude of *Halimeda*-seagrass interactions^12,50^. Our results show that *Halodule* populations are likely to withstand intermediate OA scenarios, yet local irradiance, temperature and nutrient conditions may very well play a determinant role in the magnitude of the metabolic interactions between seagrasses and sympatric calcifying macroalgae. Interspecific variations in seagrass photosynthesis due to diverse DIC assimilation mechanisms will also put some species at an advantage over others^17,51^. This was observed at volcanic CO_2_ vent sites, where seagrass community composition shifted according to seawater pH^52^. The extent to which populations are acclimated to elevated conditions may determine their long-term resilience.

Acidified seawater is ultimately unfavourable for *H. cuneata* calcification, however we demonstrate that high-performing primary producers such as *H. wrightii* are capable of providing significant refuge for these calcifying algae via biologically altering seawater chemistry. Previously, a 1.6-fold increase in the calcification rate of *Halimeda renchii* was observed in seagrass beds at ambient CO_2_ levels^13^. In our study, it was unexpected that seagrass presence did not also increase *H. cuneata* calcification at ambient CO_2_. Based on the high GPP of *H. wrightii* observed at ambient CO_2_, one would expect the consequent IUS carbonate chemistry to be exceptionally favourable and to stimulate calcification. Regardless, the issue is that future oceans will possess a much higher pCO_2_ than that of today’s oceans. Our results show that under OA, the presence of seagrass will likely foster calcification rates during the day that are comparable to those at current pCO_2_. Recent findings anticipate, however, that other factors of different functional scales will cause variation in this buffering capacity. There are often other marine macrophytes coexisting with *Halimeda* and seagrass, namely macroalgae. Benthic community composition is known to alter seawater chemistry at different magnitudes^53,54^, greatly due to species-specific irradiance optima and CCM mechanisms, therefore influencing the community’s OA buffering capacity. Modeled projections incorporating effects of OA and net community metabolism (NCM) on carbonate chemistry in seagrass meadows predict long-term offsets of CO_2_, but also NCM-driven extremes in carbonate chemistry under OA^55^. In particular, future pH levels at night are expected to be extremely low due to the intensified effect of OA on respiration^55^. This has implications for the net calcification of *Halimeda* that we weren’t capable of addressing and would need to be analysed in future studies. Additionally, Cyronak *et al*. (2018) reveal that the spatial and short temporal variation of carbonate chemistry in seagrass beds can be even greater than diel variability, thus potentially impacting the buffering capability of seagrasses across even smaller scales^56^. The fate of calcifying algae under OA may very well lie in the composition of the accompanying photoautotroph community as well as their associated NCM dynamics^54^.

Solid predictions of whether and which calcifying algae will adapt to OA & temperature rise are generally still insufficient^57^, partially due to the lack of incorporation of species interactions effects and natural seawater variability. Most of the available information produced until now has been based on stable values of pH and temperature^37^ and few global studies exist that address marine plant interactions alongside OA and temperature rise^58^. Despite the academic value of these efforts, their utilization in depicting future scenarios should be considered with caution, since natural variability of physical/chemical conditions is a selective pressure and a major driver of marine ecosystem functioning. Likewise, although there are known limitations to not manipulating CO_2_ directly into each experimental tank^59^, our design was chosen as the most feasible, which gave priority to the incorporation of diel pH and CO_2_ variability. Each tank was an isolated experimental unit with a certain degree of intrinsic variability and our results do not suggest that our design has biased the experimental outcome.

The degree of physiological tolerance or increased performance to changes in CO_2_ and temperature in the marine environment can be due to trans-generational plasticity, phenotypic buffering, or plasticity within generations (or ‘classical’ plasticity) from which ‘true’ evolutionary adaptation may arise^6^. Data supports that genetic variation in traits important for OA and temperature rise is prevalent in near-shore plants^6^. Based on our results, we suggest that the large natural variability of temperature and CO_2_ in shallow coastal environments has selected for phenotypic plasticity and co-evolutionary tools involving the metabolic interaction between *H. cuneata* and *H. wrightii*, thus potentially providing resilience and adaptability to OA. Plasticity in response to OA and temperature rise will help maintain population resilience under changing environments^60^. If *Halimeda* species have adequate genetic variability to generate phenotypes with different CO_2_ tolerances and optima, then it is likely that inter or intraspecific variability in fitness will be observed, where OA winners are likely to be those coexisting with seagrasses. The responses we observed are a contribution to the understanding of possible shifts in composition of relevant communities^61^, but they also highlight the relevance of coastal plant metabolic interactions as a dynamic biological factor that should be considered in the management of natural habitats, namely marine protected areas, in view of future climate scenarios.

## Methods

### Study area and experimental design

The experiment was performed in a large-scale, flow-through mesocosm designed by Projeto Coral Vivo, located at its research station on Araçaípe beach, Bahia, Brazil. The mesocosm was designed to test the effects of ocean acidification and warming (among other factors) on reef organisms^62^, while closely mimicking the adjacent reef conditions. Aracaípe beach’s (16° 29′ 28.6′′ S 39° 3′ 58.4′′ W) fringing reef develops 100 m off the coast of the Marine Mesocosm, where the seagrass *H. wrightii* and the upright calcifying green alga *H. cuneata* coexist. The open flow and proximity of the mesocosm to the fringing reef make this system highly realistic since it is able to maintain experimental conditions (seawater composition, temperature, diel pH and CO_2_ variability, turbidity, salinity, plankton density, photoperiod, rainfall, irradiance…etc) that are very similar to those in the adjacent reef.

With the interest of simulating moderate predictions of ocean acidification and warming, levels of pCO_2_ and temperature were chosen based on the IPCC RCP 6.0 scenario^63^ and modeling of future atmospheric emissions^64,65^. We initially established a full factorial design of ambient temperature and pCO_2_ and elevated temperature and pCO_2_, totaling four treatments. The ambient levels were the unaltered present temperature (28 °C) and pCO_2_ (380 µatm) of seawater from the adjacent reef, and the elevated CO_2_ and temperature treatments were achieved by manipulating seawater to target +2 °C (30 °C) and +0.25 pH, or +442 µatm (822 µatm). The pCO_2_ was calculated for each treatment using the TA and mean pH values via CO2SYS^66^.

Seawater from 500 m offshore was continuously pumped into four 5,000-L underground sumps where the four CO_2_ and temperature treatments were applied. pCO_2_ was manipulated in two sumps using a custom reactor system that introduced fine bubbles of CO_2_ into constantly mixed seawater. Similarly, a 1.9 m 15,000 W heater was placed in each of the two underground sumps where seawater temperature was to be elevated. Seawater pCO_2_ and temperature were not fixed. We used a custom-made Reef Angel Open-Source Controller, which elevated and regulated pCO_2_ and temperature levels with respect to ambient fluctuations. Treatments were applied to header sumps and not directly to experimental tanks based on feasibility and limitation of resources. Mixed treatment water was fed to four 310-L reservoir tanks, from which flow was regulated to 16, fully randomized 130-L raceway experimental tanks (n = 4 per treatment). Experimental tanks were continuously supplied with seawater at a flow rate of ~10 L•min^−1^, achieving a renewal rate of 5 x per hour. The experimental tank area was covered in shade cloth to uniformly reduce the intensity of natural sunlight by 70%, simulating the amount of incident solar radiation measured at approximately 2 m where organisms were collected on the reef. Duarte *et al*. (2015) reported a complete description of the mesocosm design and functioning.

### Sampling

Approximately 160 specimens of *H. cuneata* and 1,500 shoots of *H. wrightii* were collected at a depth of 2 m using SCUBA by carefully removing the entire holdfast and rhizome, respectively, and were brought to the holding aquariums of the Marine Mesocosm for sorting and removal of epibionts. Sediment from the first 10 cm of the sampling area was also collected and used as substrate for subsequent planting in the mesocosm. Ten *H. cuneata* thalli were placed upright in a plastic tray (40 × 17 × 4 cm), with the holdfasts anchored in 3 cm of sand. One tray was placed in each of the 16 experimental tanks. Approximately 50 seagrass shoots were replanted in each of 2 plastic trays with 3 cm of sediment in each experimental tank. Each of the 16 experimental tanks thus possessed 3 trays, 1 with *H. cuneata* and 2 with *H. wrightii*. Organisms were acclimated at ambient temperature and pCO_2_ for 15 days. Treatments commenced upon completion of the acclimation period, reaching target levels within 24 hours and were applied for a total of 10 days.

### Abiotic parameters

Salinity (Refractometer: Instrutherm RTS-101ATC), dissolved oxygen & temperature (Portable dissolved oxygen meter: Instrutherm MO-900), incident irradiance (Quantometer: apogee MQ-200) and pH (pHmeter: Gehaka ISO 9001) were measured daily in each experimental tank. Handheld pH meter and pH sensors were calibrated to NBS buffers daily and sensor drift was checked weekly using a bench top Gehaka pH meter. The remaining abiotic parameter meters were calibrated as per recommended in their factory manuals, using appropriate calibration solutions. The daily average photosynthetically active radiation (PAR) was monitored with light loggers (HOBO), which were positioned underwater at the level of the organisms in the experimental tanks. Nutrient concentrations were monitored every 3 days in each tank^67^. For the monitoring of seawater carbonate chemistry, water samples were retrieved from each experimental raceway tank (n = 3) and were immediately refrigerated. Total alkalinity measurements, were performed using a custom USB4000 spectrophotometer (Ocean Optics, Dunedin, USA) and compared to certified reference material (Scripps Institute of Oceanography, USA).

### Primary production and calcification

In order to isolate and assess the potential metabolic interactions between *H. cuneata* and *H. wrightii*, short-term (2.5 h) incubations were administered at the beginning and end of the experiment. Incubations were conducted on each species separately, as well as with the two species together (n = 3 per species/combination). Oxygen evolution and change in total alkalinity were measured; the former was used to calculate gross primary production (GPP) and the latter, for calcification rates and change in dissolved inorganic carbon (ΔDIC). GPP and ΔDIC were calculated for each species, whereas calcification was only calculated for the alga. An irradiance of 750 µmol quanta m^−2^ s^−1^ was used during the incubations since this was the average midday irradiance during the time period when the incubations were conducted.

Incubations did not take place in the experimental raceway tanks. An incubation setup was constructed outside of the experimental tanks using 28-L boxes, impermeable plastic bags as chambers and an illuminator. The illuminator structure was equipped with four metallic vapor lamps (220 V, REV426A4, Serwal) and positioned over four independent dark 28L boxes (22.5 × 35 × 50 cm, Marfinite). Each box was connected to an individual raceway experimental tank by a 12 mm (diam.) hose so that seawater was constantly renewed in the box. Thus, each box corresponded to a treatment (Supplementary Material Fig. S1). In each box, four transparent 29 × 39 cm incubation bags impermeable to dissolved oxygen served as incubation chambers and were filled with approximately 500 ml of seawater. Three of the bags received the following biological material: (1) only 1–2 *H. cuneata* thalli, (2) 1–2 *H. cuneata* thalli and 10 shoots of *H. wrightii* or (3) only 10 shoots of *H. wrightii* (illustrated in Supplementary Material Fig. S1). The fourth bag contained only seawater in order to monitor any background changes in oxygen concentration and total alkalinity (A_T_) due to microorganisms. Water samples were taken directly from treatment boxes for initial measurements of dissolved oxygen concentration (DO) and A_T_ immediately prior to commencement of incubations. All visible air bubbles were removed from the bags before their sealing. At the end of the incubation period, water samples were taken from each bag for the final measurements of DO and A_T_. Water volume was measured and *H. cuneata* and *H. wrightii* were removed, dried at 60 °C and weighed. Basal segments were removed from each sample, decalcified with nitric acid (0.6M HNO_3_) and weighed to determine the dry decalcified weight.

### Oxygen evolution

Five initial water samples (12 ml) were collected from each treatment box (n = 3) at the beginning of the incubation. At the end of the incubations, five water samples were taken from each bag (n = 3) with a 60 ml syringe fitted with a small tube. Samples were immediately treated with manganese chloride and alkaline-iodide reagents upon removal and refrigerated for 72 hours. They were then treated with a sulfuric acid reagent and analysed spectrophotometrically according to the Winkler method adapted by Labasque^68^ in order to calculate the dissolved oxygen concentration, or O_2_ production.

GPP values were calculated by normalizing O_2_ production to incubation time, volume of water and the decalcified dry weight of the incubated tissue (µmol O_2_ gDW^−1^ h^−1^), after removing background O_2_ fluctuations due to microbial activity.

### Calcification

One initial water sample (180 ml) was collected from each treatment box pre-incubation and one final water sample was taken from each bag post-incubation (n = 3). Water samples were immediately refrigerated until analysis. Alkalinity anomaly measurements were performed using the aforementioned Ocean Optics equipment. The CO2SYS program^66^ was used to calculate all DIC species and components of seawater carbonate chemistry. Changes in each DIC species (ΔHCO_3_^−^, ΔCO_2_ and ΔCO_3_^2−^) and total DIC (ΔDIC) were calculated by subtracting the pre-incubation value from the post-incubation value.

Calcification rates were calculated for *H. cuneata* using the following equation^69^:

1 $$g=-0.5\,\frac{{\rm{\Delta }}{A}_{T}V}{DW{\rm{\Delta }}t}$$

where g = µmol CaCO_3_ g^−1^ h^−1^, ΔA_T_ = change in total alkalinity, V = volume of incubated seawater, DW = dry, decalcified weight of *H. cuneata* and Δt = incubation time (h).

### Statistical analysis

A three-way analysis of variance (ANOVA), was performed on the *H. cuneata* calcification and GPP data (log(x + 1) transformed), with the factors CO_2_ (two levels), temperature (two levels), and seagrass presence (two levels). A two-way ANOVA was used to test *H. wrightii* GPP data (log(x + 1) transformed), with the factors CO_2_ and temperature (two levels each). Significance level was set at *p* = 0.05. Due to the absence of any temperature effect and a strong trend in the data with respect to CO_2_, we proceeded to pool the samples from the same CO_2_ treatment for a more robust analysis and in order to preserve important ecological implications. All data passed assumptions of normality of residuals and homogeneity of variances. Subsequently, two-way ANOVAs were applied to *H. cuneata* calcification, GPP data (log(x + 1) transformed), ΔHCO_3_^−^, ΔCO_2_, ΔCO_3_^2−^, ΔΩAr, ΔpH and Δtotal DIC data (log(x + 1) transformed), with the factors CO_2_ (two levels) and seagrass presence (two levels). One-way ANOVAs were applied to *H. wrightii* GPP data (log(x + 1) transformed), ΔHCO_3_^−^, ΔCO_2_, ΔCO_3_^2−^ and Δtotal DIC data (log(x + 1) transformed), with CO_2_ (two levels) as a factor. Fisher’s LSD post hoc tests were used for pairwise comparisons of significant effects. All statistical analyses were performed using IBM SPSS Statistics 24.

## Electronic supplementary material

Supplementary material

## Data Availability

The datasets generated during and/or analysed during the current study are available from the corresponding author upon reasonable request.
