# Publisher Correction: Seagrass can mitigate negative ocean acidification effects on calcifying algae

**DOI:** 10.1038/s41598-020-66683-6

**Published:** 2020-06-12

**Authors:** Ellie Bergstrom, João Silva, Cíntia Martins, Paulo Horta

**Affiliations:** 1 School of Environment & Science and Australian Rivers Institute – Nathan Campus, Griffith University, 170 Kessels Road, Brisbane, Nathan, Queensland 4111 Australia; 2 Laboratory of Phycology, Department of Botany, Center for Biological Sciences, Federal University of Santa Catarina, 88040-970 Florianópolis, SC Brazil; 3 CCMar - Centre of Marine Sciences, University of Algarve, Campus of Gambelas, 8005-139 Faro, Portugal; 4 Department of Ecology and Zoology, Center for Biological Sciences, Federal University of Santa Catarina, 88010-970 Florianópolis, SC Brazil

**Correction to:** *Scientific Reports* 10.1038/s41598-018-35670-3, published online 13 February 2019

In the original version of this Article, Cíntia Martins was incorrectly listed as a second corresponding author. The correct and only corresponding author for this Article is Ellie Bergstrom. Correspondence and request for materials should be addressed to ellie.bergstrom@griffithuni.edu.au.

Further to this, the original version of this Article omitted an affiliation for Ellie Bergstrom. The correct affiliations for Ellie Bergstrom are listed below:

School of Environment & Science and Australian Rivers Institute – Nathan Campus, Griffith University, 170 Kessels Road, Brisbane, Nathan, Queensland, 4111, Australia

Laboratory of Phycology, Department of Botany, Center for Biological Sciences, Federal University of Santa Catarina, 88040-970 Florianópolis, SC, Brazil

These errors have now been corrected in the HTML and PDF versions of the Article, and in the accompanying Supplemental Material.

