# Supplementary material for: Seagrass can mitigate negative ocean acidification effects on calcifying algae
Source: Sci Rep. 2019 Feb 13;9:1932. doi: 10.1038/s41598-018-35670-3 (PMC6374406; doi:10.1038/s41598-018-35670-3)
Supplement: Supplementary file 1 — Supplementary material [file 41598_2018_35670_MOESM1_ESM.pdf]

Supplementary material

Manuscript title

Seagrass can mitigate negative ocean acidification effects on calcifying algae

Author list and affiliations

Ellie Bergstrom[1*]	ellie.bergstrom@griffithuni.edu.au
João Silva[2]	jmsilva@ualg.pt
Cíntia Martins[3]	cintia_ufpr@yahoo.com.br
Paulo Horta[3]	paulo.horta@ufsc.br

[1]School of Environment & Science and Australian Rivers Institute – Nathan Campus, Griffith University, 170 Kessels Road, Brisbane, Nathan, Queensland 4111, Australia
[2]CCMar - Centre of Marine Sciences, University of Algarve, Campus of Gambelas, 8005-139 Faro, Portugal
[3]Department of Ecology and Zoology, Center for Biological Sciences, Federal University of Santa Catarina, 88010-970, Florianópolis, SC, Brazil
*corresponding author

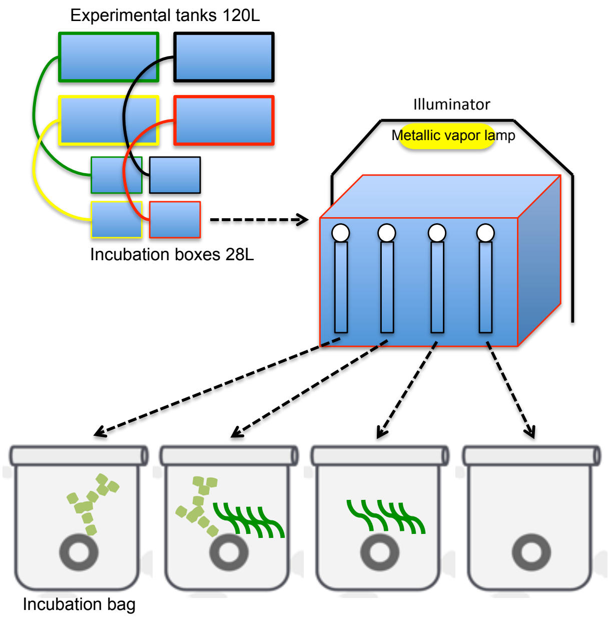

Fig. S1. Incubation and illuminator design for the incubation of *H. cuneata* alone, *H. wrightii* alone and both together under 4 CO_2 and temperature treatment combinations. Each tank/box colour represents a different treatment.

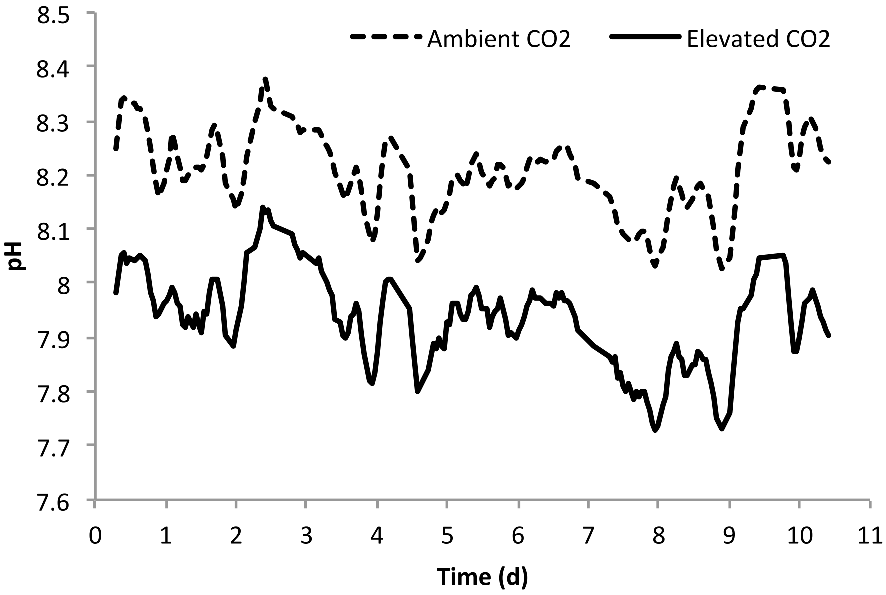

Fig. S2. pH fluctuation throughout the experimental period for the ambient and elevated pCO_2 treatments.

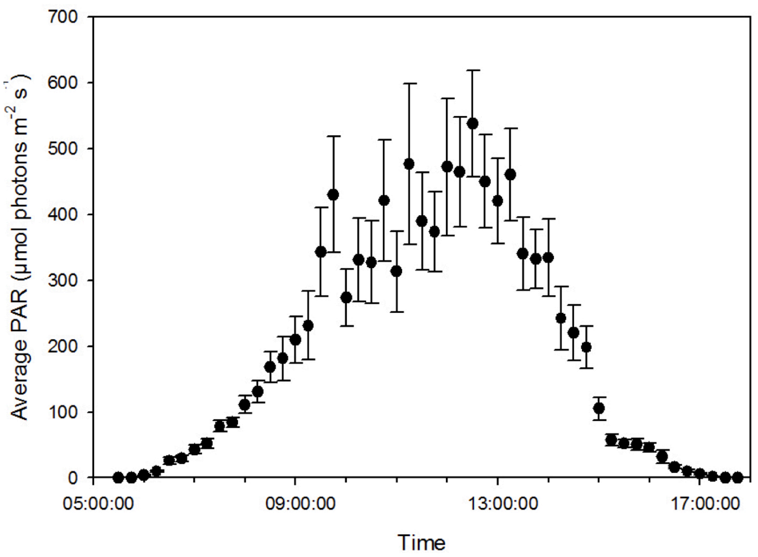

Fig. S3. Mean daily PAR (µmol photons $m^{-2}s^{-1}$) throughout the experiment ± SEM.
